# Generation of restriction endonucleases barcode map to trace SARS-CoV-2 origin and evolution

**DOI:** 10.1038/s41598-021-91264-6

**Published:** 2021-06-03

**Authors:** Federico Colombo, Elisa Corsiero, Myles J. Lewis, Costantino Pitzalis

**Affiliations:** Centre for Experimental Medicine and Rheumatology, William Harvey Research Institute, Barts and The London School of Medicine and Dentistry, Queen Mary University of London, John Vane Science Centre, Charterhouse Square, London, EC1M 6BQ UK

**Keywords:** Viral infection, Molecular biology, Genetic mapping

## Abstract

Since the first report of SARS-CoV-2 in China in 2019, there has been a huge debate about the origin. In this work, using a different method we aimed to strengthen the observation that no evidence of genetic manipulation has been found by (1) detecting classical restriction site (RS) sequence in human SARS-CoV-2 genomes and (2) comparing them with other recombinant SARS-CoV-like virus created for experimental purposes. Finally, we propose a novel approach consisting in the generation of a restriction endonucleases site map of SARS-CoV-2 and other related coronavirus genomes to be used as a fingerprint to trace the virus evolution.

## Introduction

Coronaviruses (CoVs) goes into the family *Coronaviridae* causing symptoms primarily in the upper respiratory tracts which range from common cold to severe to fatal illnesses^1^. They have been associated with two major disease outbreaks, the severe acute respiratory syndrome (SARS-CoV, 2002) and the Middle East respiratory syndrome (MERS-CoV, 2012)^2^. In December 2019, a new coronavirus (SARS-CoV-2) started to cause viral pneumonia bringing to severe and fatal infection. Although SARS-CoV-2 belongs to the same lineage of CoVs that causes SARS, it is genetically different and it cluster apart exploiting phylogenetic trees^3^. Phylogenetic analysis demonstrated the highly similarity between human SARS-CoV-2 and the sequence isolated from the Bat-Cov-raTG13^4^ (97.2% identity) and the Pangolin-SARS-CoV^5^ (80% identity), particularly in the receptor-binding-domain (RBD) of the S protein, important to mediate binding to human-receptor-angiotensin-converting-enzyme-2 (hACE2)^6^. The World Health Organization declared a coronavirus disease 2019 (COVID-19) pandemic in March 2020. Therefore, one of the major discussions around SARS-CoV-2 has been related to its origin with the assumption that SARS-CoV-2 could have been the result of genetic manipulations or spill-over from laboratories studying these viruses. In March 2020, Anderson and colleagues published a detailed analysis showing that SARS-CoV-2 does not derive from a laboratory construct^7^. Although other several coronavirus experts have discredited the hypothesis of a man-made coronavirus^8–12^, here we aim to present a different method based on the analysis of restriction site (RS) sequences in the genome of SARS-Cov-2 to reconstruct its origin and follow the new variants.

## Results and discussion

### What restriction sites (RS) sequence of the viral genome can say: generation of a restriction endonucleases barcoding map

**Figure 1:**
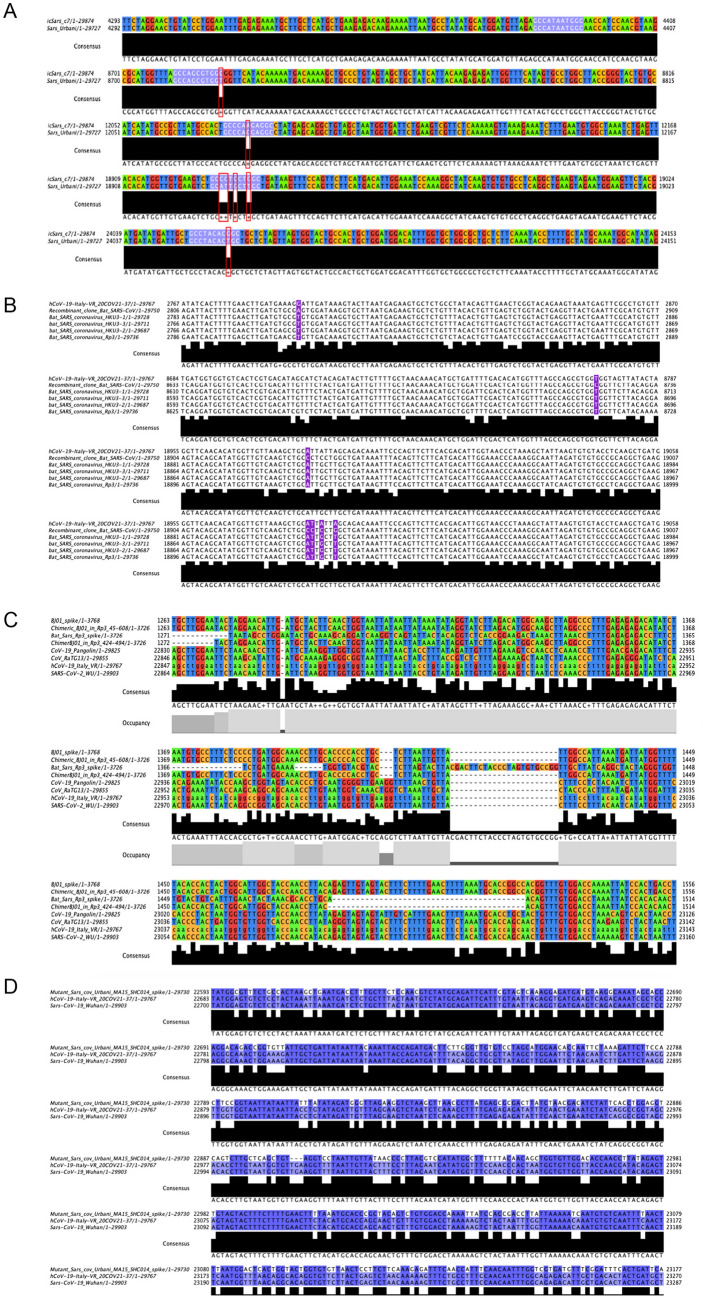
Analyses of several RSs sequences in natural and recombinant viruses. (**A**) Alignment between the WT SARS-CoV Urbani and the icSARS-CoV. The violet box highlight BglI RSs used to build the recombinant icSARS-CoV. The red boxes show the different nucleotides present in the wilt type SARS-CoV Urbani. (**B**) This alignment shows in violet specific markers used to build a recombinant spike between the Bat-SCoVs genomes HKU3 and RP3. In the hCoV-19-Italy-VR sequence most of these markers’ sites are not present, while are similar to the wilt type virus HKU3 and RP3. (**C**) Multiple sequence alignment performed with ClustalW and visualised with JalView show the poor similarities in the RBD between chimeric Spikes generated in the laboratory (line 2 and 4) compared with other SARS-CoV sequences. Despite some small regions are conserved the chimeric spikes show single bp mutation (substitution, deletion, insertions) which support natural evolutions instead of man-made manipulation. (**D**) A specific area of the alignment performed between the mutant SARS-CoV-Urbani MA15 containing the SHC014 spike with the hCoV-19-Italy-VR and the SARS-CoV-19 Wuhan. Also, in this case, the recombinant virus shows several nucleotide mutations which exclude the manipulations performed using modified primers and unique restriction sites.

During the SARS-CoV epidemic outbreak in 2003, a method called reverse genetic to assemble a full-length cDNA of the SARS-CoV-Urbani strain, as a template for manipulation of the viral genome, was published to develop and test candidate vaccines and therapeutics^13^. This resulted in the so-called infectious clone icSARS-CoV containing atypical markers of the wild-type (WT) virus. In particular, several *Bgl*1 RSs were introduced into the icSARS-CoV cDNA, which can be recognized since mutation are included in the newly formed cDNA. Figure 1A shows the sequence alignment between the WT SARS-CoV-Urbani and the icSARS-CoV. We highlighted the sequence containing the *Bgl1* RS used to produce icSARS-CoV.

The newly sequences introduced in the recombinant cDNA of SARS-CoV can be used as markers to follow possible virus laboratory spillage. We analysed natural sequences isolated from four different SARS-CoV (hCoV-19-Italy-Vr/hSARS-CoV-19-Wuhan/hCoV-19 Pangolin/Bat-Cov-raTG13) to look for *Bgl1* RS ‘marker’ (GCCNNNN/NGGC). All the genomes did not contain these sites (Table S1), in particular, the hSARS-CoV-19-Wuhan and hCoV-19-Italy-Vr.

By analysing the sequences of Bat-CoV-raTG13 and the hCoV-19-Pangolin, we observed that only one sequence from SARS-CoV-Urbani (GCCAGCGTGGT) was found in SARS-CoV-2Wu (Wuhan). This is expected since the first part of these two genomes show high similarities.

Another recombinant SARS-CoV was produced in 2007 which derived from fifteen passages of the SARS-CoV-Urbani in BALB/c mouse lungs, therefore it was named Mouse-Adapted (MA)-SARS-CoV^14^. The identity of MA-SARS-CoV compared with the original SARS-CoV-Urbani is 99.97% with only six distinct nucleotides, that cannot be used as markers of this recombinant virus since same mutations are naturally acquired by the WT-SARS-CoV, as demonstrated from the sequences of other isolated SARS-CoV^14^. Both the icSARS-CoV and the MA-SARS-CoV have become the most widely used recombinant viruses to study SARS-like viruses and no specific sequences were found in the human SARS-CoV-2Wu.

In 2008, a consensus sequence called Bat-SCoV (FJ211859) was generated starting from four Bat-SCoVs genomes HKU3–1 (DQ022305), HKU3–2 (DQ084200), HKU3–3 (DQ084199), and RP3 (DQ071615)^15^. The full-length Bat-SCoV infectious clone, generated with the method described by Yount et al.^13^ include in the recombinant sequence specific markers such as the *Bgl*1 RSs. These RSs have specific nucleic base pairs in the “N” positions of the recombinant Bat-SCoV (see supplementary Fig. 5 of Becker et al*.* summarizing all the markers found^15^). We observed that these specific sequences were all absent (Fig. 1B, Figure S1).

### Other markers to identify the origin of SARS-Cov-2

In 2008, Ren et al.showed that SARS-like coronavirus (SL-CoVs) from horseshoe bat, which has a high similarity to SARS-CoV, differed in the N-terminus of the spike protein and particularly in the receptor binding RBS region^16^. Therefore, SL-CoVs were not able to infect hACE2 expressing cells, but only chimeric viruses expressing the spike protein of the SARS-CoV were able to bind the hACE2 which is the functional receptor of SARS-CoV. The authors identified a specific region responsible for the virus entrance into hACE2-expressing cells consisting of a minimal region of less than 200 amino acids. Interestingly, this group showed that chimeric spike proteins, whereby different regions of the SARS-CoV BJ01 (BJ01-S) spike were substituted into the spike of the bat SL-CoV (Rp3), and able to bind the hACE receptor. We generated in silico two of this chimeric spike (CS) sequences (the CS_424-494_ and the CS_45-608_), and then performed a multiple alignments to check similarities between other spikes identified after 2008, including the Bat-Cov-raTG13, the hCoV-19-Pangolin and the human SARS-CoV-2. The similarities of these two chimeric spikes are limited in the RBD of the spike (Fig. 1C) and in the polybasic cleavage site (Figure S2). Thus, the recombinant spike as possible progenitors of the hSARS-CoV-2 spike sequence can be excluded.

Moreover, we performed a nucleotide blast sequence to find whether these recombinant spikes are found in the recently identified SARS-CoV-2 viruses. As shown in Figure S3–S5, we observed that, despite high similarities, many gaps (intended as single base mutations) are present between WT viruses and the recombinant spikes.

The turning point arrived in 2013, when Xing-Yi and colleagues published an important paper showing that a WT bat SL-CoV was capable of using hACE2 as an entry receptor, dispelling the observation that no natural SL-SARS-CoV were able to use hACE2. Interestingly, the newly identified bat SL-CoV-WIV1 had high sequence similarity (99.9% identity) to two other identified WT bat coronaviruses, RsSHC014 and RS3367. This study suggested that direct bat-to-human infection is a possible scenario for some bat SL-CoVs. In 2015, Vineet et al.made a recombinant virus between the spike of the bat coronavirus SHC014 and the mouse-adapted SARS-CoV backbone^17^ using the well establish reverse genetic approach^13^. According to this method, several *Bgl*1 RSs were included into the sequence (Table S2). Moreover, the sequences between the newly mutant SARS-CoV have poor sequence similarity to human SARS-CoV-19-Italy-VR and the SARS-CoV-19-Wuhan (Fig. 1D).

### Unique restriction sequence sites: a novel approach to track the SARS-CoV-2 origin

**Figure 2:**
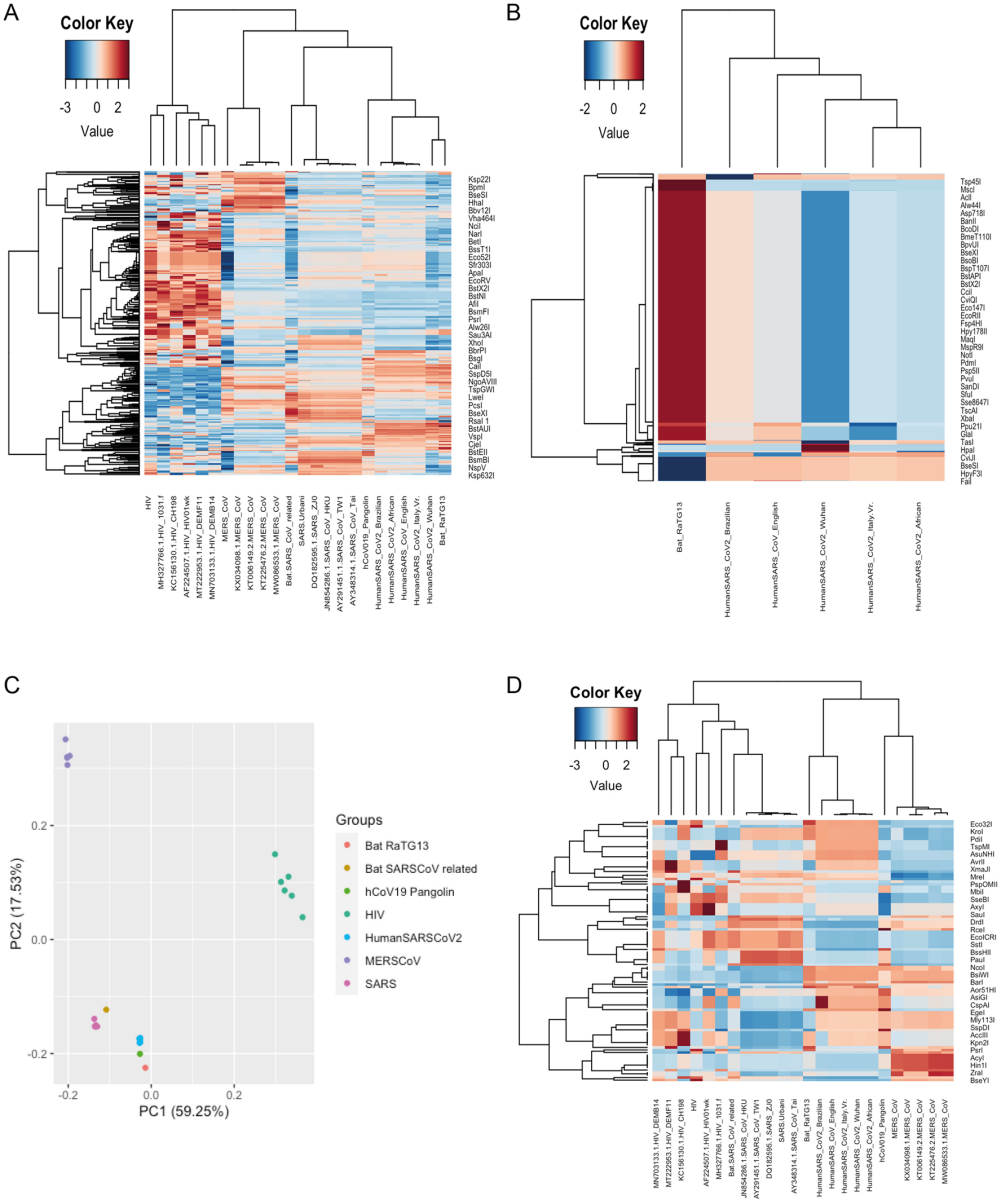
RS barcodes map help to determine the distance between different viruses’ genomes. (**A**) RSs barcode map of 7 different virus genomes. The colour scale represents the frequency to find that specific RS in the genome after using the Serial Cloner Library using 741 RSs. The hierarchical clustering was performed using Pearson correlation as distance metric. It is clear that some barcode patterns highlight similarities between related viruses, while other patterns show dissimilarities, such as the case of the HIV, used as control, which shows a clear different barcode compared with SARS-CoV-2 genomes. B) RSs barcode map performed on a region of 300 bp previously identified from the full map. Here, we compared the two human SARS-CoV-2 (Wuhan and Italian-VR) with most closed genome of the Bat-RaTG13. The barcode generated easily highlights similarities or differences between genomes presenting high genetic similarities. (**C**) Principal component analysis (PCA) plot generated with the frequencies of the RSs, retrieved from each of these genomes, confirms same distance between viruses’ genomes. (**D**) Barcode Heatmap with hierarchical clustering based on the most informative RSs showing that Human SARS-CoV-2 and Bat-Cov-RaTG13 are evolutionarily closer than hCoV-19 Pangolin and MERS CoV. The clustering performed with less RSs confirms that we are still able to generate the right distance metric.

Exploiting the RS sequences, which are approximately 6–8 base pairs of DNA, as specific markers, we propose an alternative way to trace the SARS-CoV-2 origin. This approach consists in the generation of a RS map of SARS-CoV-2 and the other four related coronavirus genomes. Using the Serial Cloner Restriction Enzyme Library, we generated the RS barcoding map based on the frequency of finding specific RS sequences in the genome. First, we generated a RS barcoding map which was used as genetic fingerprinting of the specific sequence analysed and which easily highlights sequence differences between the genomes. The pattern of the barcode’s reconstruction demonstrated high similarity between the coronavirus isolated from the Bat-Cov-raTG13 and the Pangolin, suggesting a natural evolution and adaptation of the virus. Different sequences of HIV, SARS-CoV and MERS-CoV were used as controls (Fig. 2A and Figure S6).

From the full restriction enzyme barcoding map, we identified in the spike (S) gene a sequence of 300 bp that can be used as a barcode to identify the virus and differentiate from others (Fig. 2B and S7). It is also important to note that this method allows small mutations between new variants to be appreciated. This approach is low-cost and does not require full sequencing of the virus genome and extended analyses conducted by bioinformaticians. Indeed, by using a standard PCR reaction to amplify the above mentioned 300 bp spike gene, or simply by using real-time PRC products from swab test, and subsequent sequencing of this region, it is possible to generate an RS barcode that will give us a low-cost system to follow viral mutation and trace it over subsequent years. This would allow more samples to be tracked, especially with the emergence of new variants (English, Brazilian, African, etc.) that may potentially be more infectious and other variants for which vaccines may be less effective^18^. Moreover, this approach can easily be used to discriminate between false negative and false positive which are the reasons for important additional socio-economic disruptions^19^.

Using the data to generate the full barcode map we performed principal component analysis (PCA) to determine whether the observed frequency of RSs is related to the hierarchical distance of the genomes analysed. The PCA plot shows a cluster formed by the Pangolin, the Bat-Cov-raTG13 and human SARS-CoV-2 (5 different sequences including the English, Brazilian, African variant and the isolated sequences in Italy and Wuhan.) (Fig. 2C). Nearby we find the cluster formed by the bat SARS-CoV related and other 5 SARS-CoV sequences. The HIV genomes and the MERS genomes were used as control and clearly clustered apart showing a greater difference in sequence identity from SARS-CoV virus.

In addition, we focalised on informative RSs to perform hierarchical clustering on the heatmap using Pearson correlation as the distance metric (Fig. 2D and S8). The heatmap confirms that human SARS-CoV-2 (all the variants) and Bat-Cov-raTG13 are closer than hCoV-19 Pangolin and MERS CoV.

Finally, the barcode map of the RSs confirmed the absence of unique sites suggesting that the SARS-CoV-19 is the product of a natural evolutionary process of single base insertions/deletions or/and recombination.

**Figure 3:**
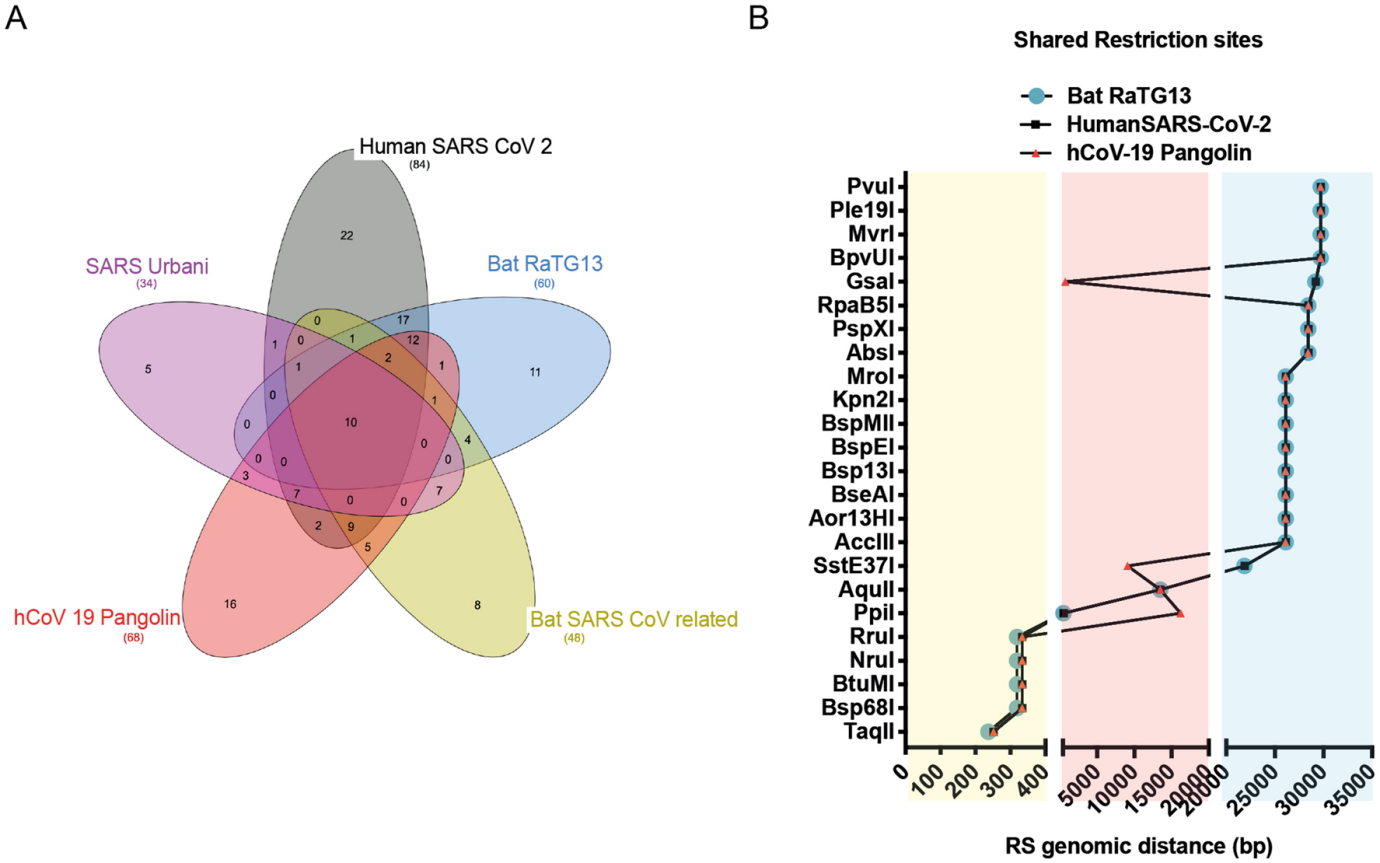
Shared restriction sites and their genomic distance. (**A**) Venn Diagram shows shared RSs between genomes of different viruses. (**B**) Genomic distance in bp of the shared RSs between SARS-CoV-Wu, Bat RaTG13 and the hCoV-19 Pangolin.

We then focused on the unique RS sequences used to modify the viral genome. In particular, we analysed shared sites between SARS-CoV-19Wu, Bat-Cov-raTG13 and Pangolin-SARS-CoV-19. Only six RS sequences were shared between these genomes and their location does not suggest their use. In the Venn diagram shown in Fig. 3A, there are 12-shared RS. However, they are only six if we consider that some of these enzymes recognize the same sequences. One example is the unique RS sequence recognized by Bsp68I, BtuMI, NruI, RruI found at 319 bp on the Bat-Cov-raTG13 and shifted at 334 bp on the SARS-CoV-19Wu and the Pangolin-SARS-CoV-19. This 15 bp shift is due to single base insertions (Fig. 3B).

Another example is the unique RS sequence GAGCTC recognized by Ecl136II on the SARS-CoV-19 genome that is located at 15081 bp, while on the Bat-Cov-raTG13 genome we found two of these sequences, one at 15080 bp and the other one at 19768 bp. The latter, if it were to be the result of genetic engineering, would be predicted to produce a gap of 6 bp, while from the local alignment it is clear that a nucleotide substitution occurred from C to T forming the new site (Figure S9A).

Finally, the genomic location of these unique sites does not flank specific ORF. Indeed, engineered RSs are typically expected to be at the beginning and at the end of an ORF. Here, all the unique RSs are located inside the ORFs (Figure S9B), thus not easily editable by conventional genetic engineering.

## Discussion

Here, we analysed the peer-review literature of the SARS-related viruses generated in the laboratory over the years used to study the evolution of Coronaviruses and to generate drugs for their treatment. We have demonstrated through the analysis of RS, that SARS-CoV-2 does not contain peculiar RS or other markers that suggest a manipulation deriving from the recombinant viruses known in the literature. Indeed, the use of RS remains today the simplest, fastest and safest way to modify and study recombinant DNA. However, it should be mentioned that other methods have been used for generating recombinant clones, such as the one called transformation-associated recombination (TAR) cloning^20^. This method has proved effective for engineering large viral DNA^21, 22^ and has only recently been developed for viruses with large RNA genomes exploiting *Saccharomyces cerevisiae*^23–25^*.* Also, bacterial artificial chromosomes (BACs) were exploited to manipulate the transmissible gastroenteritis coronavirus (TGEV) PUR46-MAD virus^26^ and although nowadays other genetic manipulation mechanisms are known that allow no traces to be left, such as the use of the Crispr-cas system^27, 28^, these remain more disadvantageous because they require higher technical capacity and higher costs and times. Furthermore, according to our knowledge, in the literature, there are no reports of SARS-CoV-2 coronavirus modifications through these more sophisticated techniques yet.

Finally, we used RS as markers to build a barcode map that could uniquely identify a particular virus. Recently, other authors used mathematical algorithms to find barcodes that identified a particular viral genome and build phylogenetic trees^29, 30^ while Son and colleagues developed a simple method to discriminate SARS-CoV-2 from SARS-CoV using one-step RT-PCR followed by restriction fragment length polymorphism^31^. Although this method, based on only three restriction enzymes, succeeds in distinguishing between SARS-CoV2 and SARS-CoV, it may not be sensitive to discriminate any mutations outside that region within the virus genome and does not allow new variants to be tracked. Guan and colleagues developed an elegant study based on the identification of a barcode that allows the SARS-CoV-2 genome to be assigned to 5 different clades, however this process requires advanced bioinformatics skills^32^. Here instead we have shown that with our method it is sufficient to sequence a region of 300 bp to build a specific barcode to distinguish the genome of a virus, included the new variants (English, Brazilian and African) and to trace its evolution over time. This would allow us to have useful information quickly and economically during the classic tests performed on swabs.

## Methods

### Genomes used for the study

SARS-CoV-2 Wuhan-Hu-1, GenBank: MN908947.3; SARS-CoV-2-English, Gisaid: EPI_ISL_816718; SARS-CoV-2-African, Gisaid: EPI_ISL_678597; SARS-CoV-2-Brazilian, Gisaid: EPI_ISL_792680; SARS-CoV-2-19/Italy/VR (Gisaid accession id: EPI_ISL_422438|2020-03-25); SARS Urbani, GenBank: AY278741.1; SARS-CoV TW1, GenBank: AY291451.1; SARS-CoV-2 HKU, GenBank: JN854286.1; SARS-CoV-2 ZJ0, GenBank: DQ182595.1; SARS-CoV-2 Tai, GenBank: AY348314.1; HIV-1, GenBank: KY580639.1, MT222953.1, AF224507.1, MN703133.1, MH327766.1, KC156130.1; MERS-CoV, NCBI Reference Sequence: NC_019843.3; MERS-CoV, GenBank: KX034098.1, KT006149.2, KT225476.2, MW086533.1; Bat SARS-like Rs4231, GenBank: KY417146.1; Pangolin-CoV, GISAID accession numbers EPI_ISL_410721; Bat CoV RaTG13 GenBank: MN996532.1.

All the genomic sequences and recombinant spikes sequences used in this study were generated following the materials and methods of the literature taken in considerations and saved in xdna format which is compatible with Serial Cloner. The files are available upon request to the authors.

### Alignments

The sequences were aligned using Serial Cloner, Blastn suite^33^, ClustalW^34^ and Jalview^35^.

### Generation of restriction enzyme barcode

The restriction enzyme map barcode of each genome was obtained using Serial Cloner library. Using this software each genome was analysed in order to obtain the frequency of each restriction site to occur in that genome. The total frequencies of all the restriction sites present in the library were used to generate the barcode map. The InteractiVenn^36^ was used to make Venn diagram.

### Genomic distance in bp between restriction enzyme sites

The genomic distance in bp between two or more restriction enzymes sites was calculated with serial cloner and then reported graphically using Prism GraphPad v8.

### Principal component analyses (PCA)

PCA analyses was performed on the frequencies of the restriction enzymes sites on the different viruses’ genomes and plotted by ggbiplot R-studio. Codes available upon request to the authors.

### Heatmap and hierarchical clustering

The heatmaps were generated in R-studio by using frequencies of the restriction enzymes sites on the different viruses’ genomes. The hierarchical clustering was performed using Pearson correlation as distance metric and Ward D clustering algorithm. Codes available upon request to the authors.

### 300 bp specific region

The 300 bp region was determined analysing the area of major discrepancy (low identity) between genomes, in particular between related genomes. This area was identified inside the spike (S) region. To generate the barcode map of these 300 bp regions we used the same method used for the full-length genomes. Thus, we calculated the frequencies of the restriction sites to generate the heatmap.

### Informative sites

As informative sites we chose all those restriction sites that showed strong discrepancy in the cut-off frequency between the various genomes. Thus, to give an example, sites that had a high cut-off frequency in genome A compared to genome B, or sites unique to genome B that are repeatedly frequent in genome A (and vice versa). Then all non-informative sites, designated as those sites equally frequent across genomes, were discarded. In total we selected 104 informative sites here listed: "AatII" "AccBSI" "AcyI" "AfeI" "AloI" "Aor51HI" "AspA2I" "AsuNHI" "AvrII" "AxyI" "BarI" "BbvCI" "BcgI" "BlnI" "BmtI" "BplI" "BsaHI" "Bse21I" "BseYI" "BsiWI" "Bsp19I" "BspOI" "BsrBI" "BssNI" "BstACI" "Bsu36I" "BtgZI" "CchIII" "Cfr9I" "Ecl136II" "Eco32I" "Eco47III" "Eco53kI" "Eco81I" "EcoICRI" "EcoRV" "GdiII" "Hin1I" "Hsp92I" "MbiI" "MreI" "NcoI" "NheI" "NmeAIII" "Pfl23II" "PfoI" "Psp124BI" "PspLI" "PspOMII" "PsrI" "RpaBI" "SacI" "SauI" "SmaI" "SplI" "Sse232I" "SstI" "TspMI" "UcoMSI" "XmaI" "XmaJI" "ZraI" "AasI" "AccIII" "AgeI" "AsiGI" "BsePI" "BshTI" "Bsp13I" "BspEI" "BspMII" "BssHII" "CspAI" "DinI" "DrdI" "DseDI" "EciI" "Eco147I" "EgeI" "FspAI" "KasI" "Kpn2I" "KroI" "KspAI" "McaTI" "Mly113I" "MroI" "MroNI" "NaeI" "NarI" "NgoMIV" "PacI" "PasI" "PauI" "PceI" "PdiI" "PinAI" "PteI" "RceI" "SalI" "SfoI" "SseBI" "SspDI" "StuI". To generate the barcode map of the informative sites we used the method described for the full length and the 300 bp region.

## Supplementary Information

Supplementary Information.

## Data Availability

The datasets and codes used and/or analysed during the current study are available from the corresponding author on reasonable request.
