# Supplementary material for: Generation of restriction endonucleases barcode map to trace SARS-CoV-2 origin and evolution
Source: Sci Rep. 2021 Jun 3;11:11773. doi: 10.1038/s41598-021-91264-6 (PMC8175367; doi:10.1038/s41598-021-91264-6)
Supplement: Supplementary file 1 — Supplementary Information. [file 41598_2021_91264_MOESM1_ESM.pdf]

Supplementary Information

Generation of restriction endonucleases barcoding map to trace SARSars-CoV-2 origin and evolution.

Federico Colombo[1†], Elisa Corsiero[1†], Myles J. Lewis[1], and Costantino Pitzalis[1]

[1]Centre for Experimental Medicine & Rheumatology, William Harvey Research Institute, Barts and The London School of Medicine & Dentistry, Queen Mary University of London, John Vane Science Centre, Charterhouse Square

[†] Corresponding Authors: Federico Colombo and Elisa Corsiero

Centre for Experimental Medicine & Rheumatology, William Harvey Research Institute, Barts and The London School of Medicine & Dentistry, Queen Mary University of London, John Vane Science Centre, Charterhouse Square

London EC1M 6BQ, UK

Tel: +44 (0)2078828193

Fax: +44 (0)2078826104

Email: f.colombo@qmul.ac.uk; e.corsiero@qmul.ac.uk

The authors declare that they have no conflict of interest.

Supplementary Figure Legends

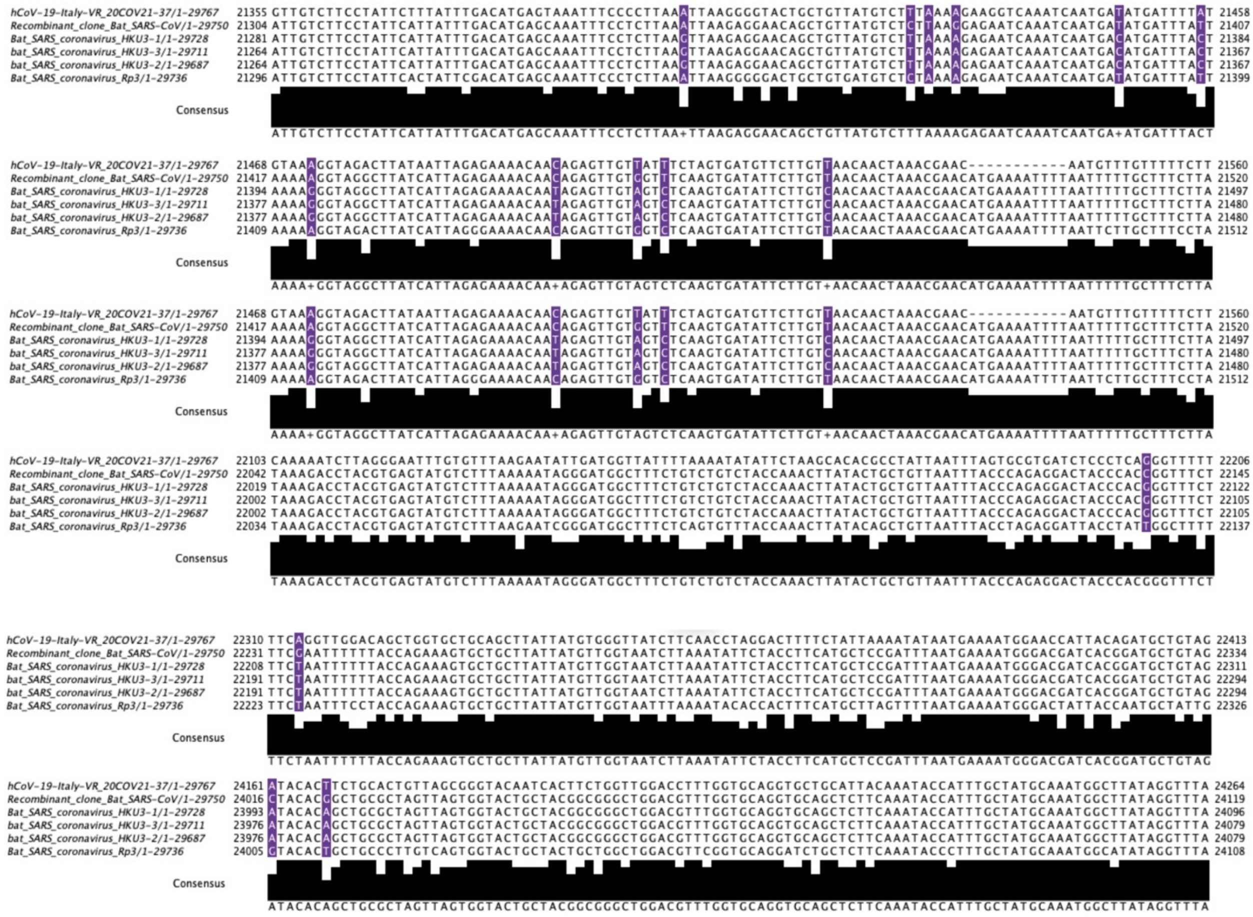

Figure S1. Specific markers used to build the full-length Bat-SCoV infectious clone. Full size of the Figure 2B that highlights in violet specific markers used to build a recombinant spike between the Bat-SCoVs genomes HKU3 and RP3. In the hCoV-19-Italy-VR sequence most of these markers' sites are not present, while are similar to the wilt type virus HKU3 and RP3.

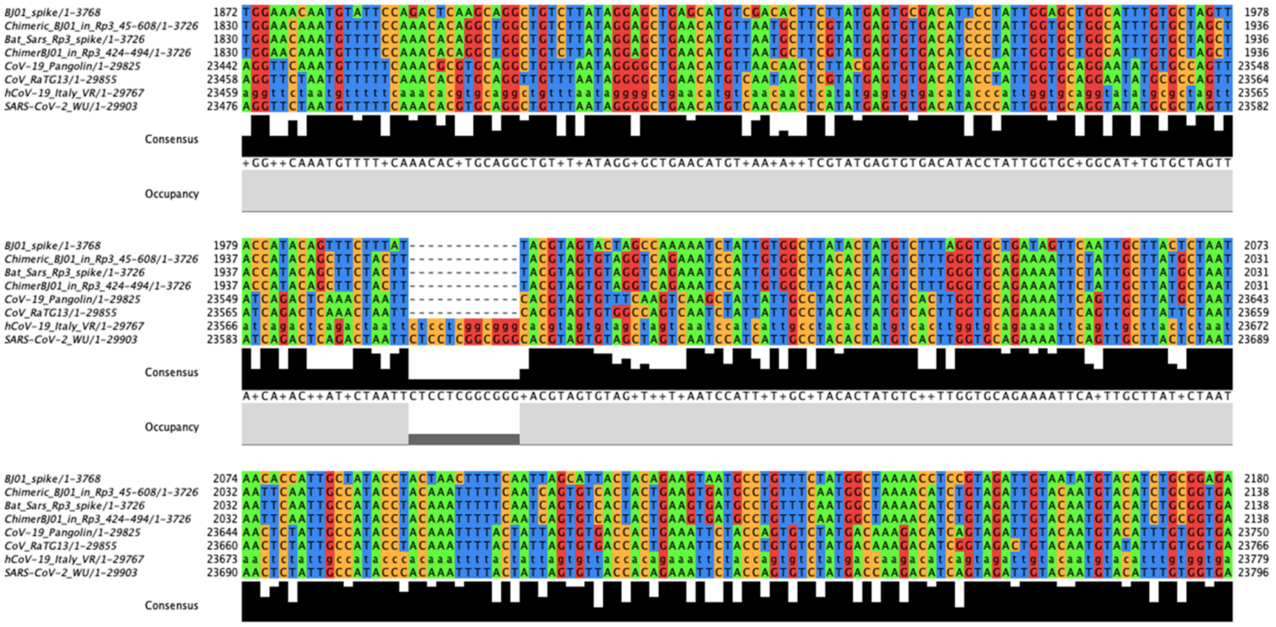

Figure S2. Polybasic cleavage site in two chimeric spikes from BJ01 and RP3. Multiple sequence alignment performed with ClustalW and visualised with JalView show the differences in the polybasic cleavage site between chimeric Spikes (BJ01 and RP3) generated in the laboratory (line 2 and 4) compared with other SARS-CoV sequences.

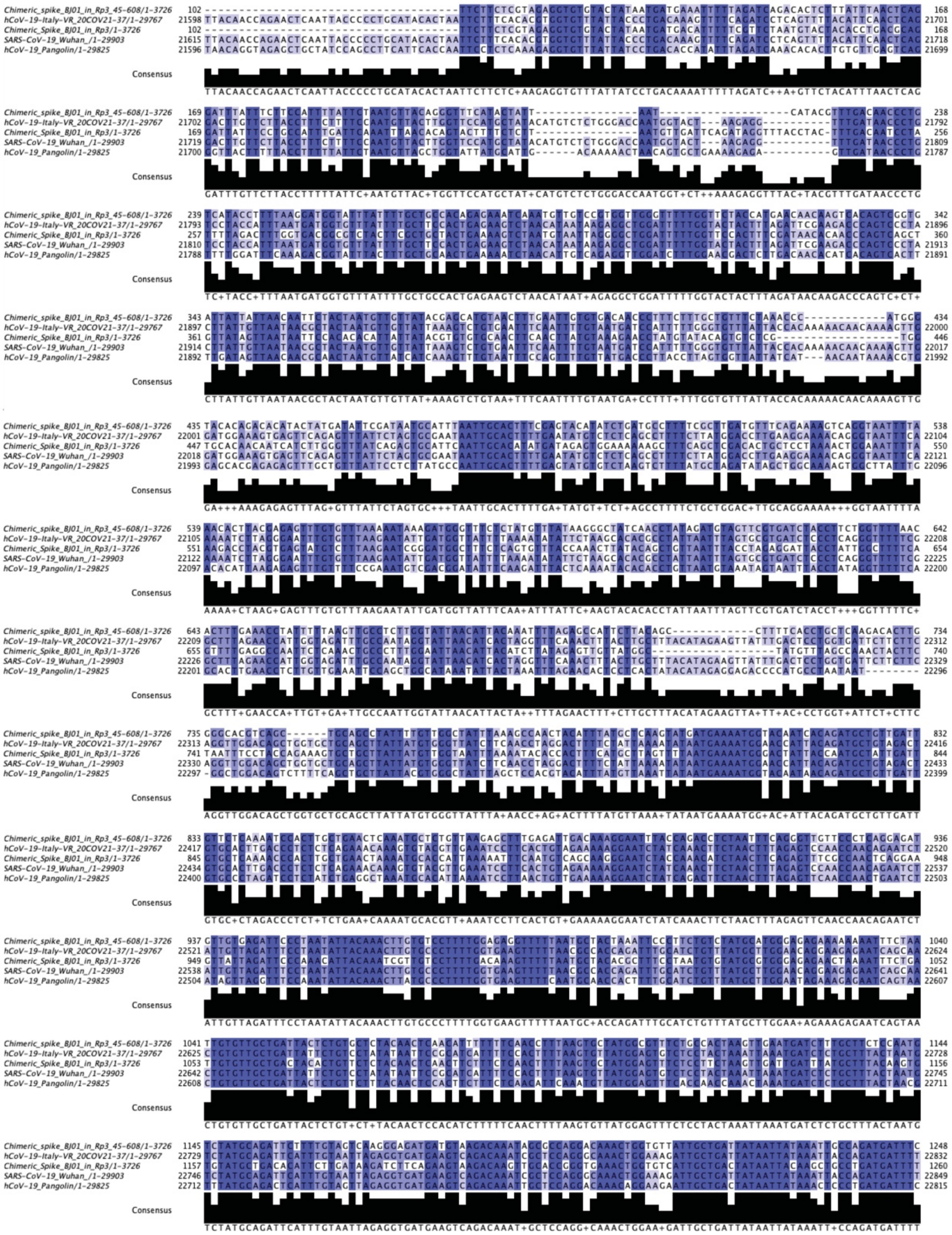

Figure S3. **Full alignment between chimeric BJ01-RP3 and other Sars-CoV-2.**

Alignment of two chimeric spikes (two different BJ01 aminoacidic sequence (424-494; 45-608) into the RP3) with the SARS-CoV 2 isolated in Italy and Wuhan and from the Pangolin.

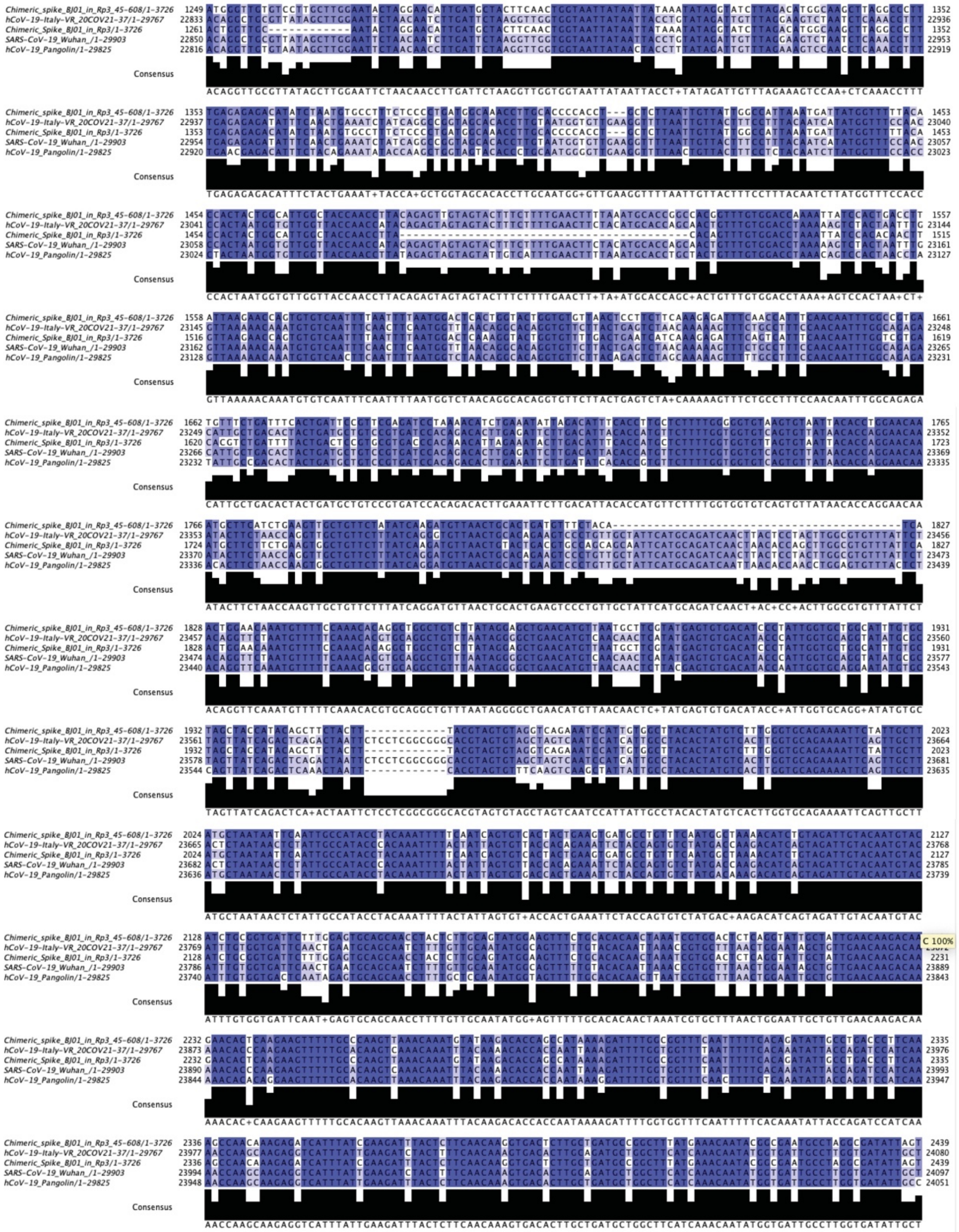

Figure S4. Full alignment between chimeric BJ01-RP3 and other Sars-CoV-2.

Alignment of two chimeric spikes (two different BJ01 aminoacidic sequence (424-494; 45-608) into the RP3) with the SARS-CoV 2 isolated in Italy and Wuhan and from the Pangolin.

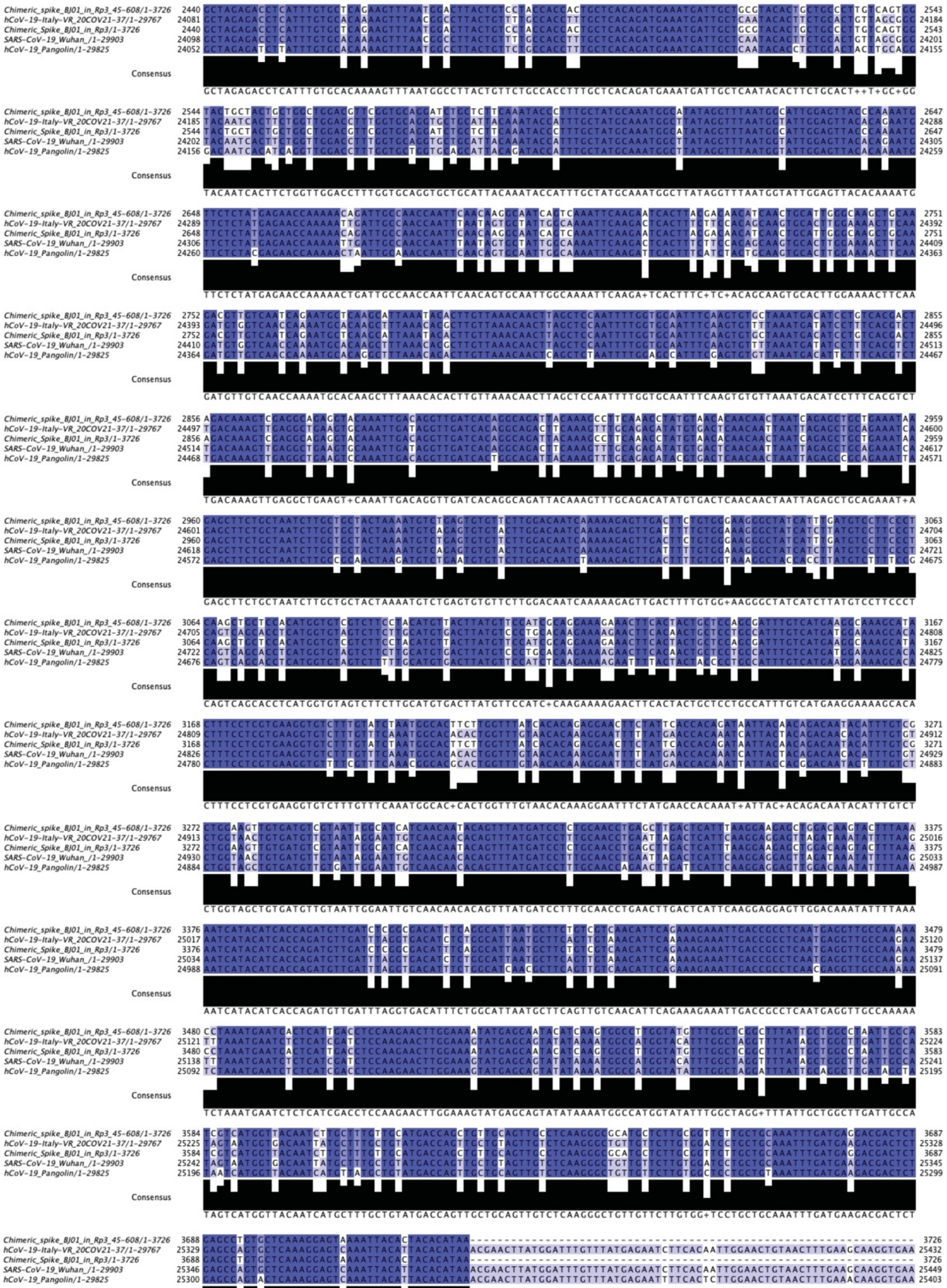

Figure S5. Full alignment between chimeric BJ01-RP3 and other Sars-CoV-2.

Alignment of two chimeric spikes (two different BJ01 aminoacidic sequence (424-494; 45-608) into the RP3) with the SARS-CoV 2 isolated in Italy and Wuhan and from the Pangolin.

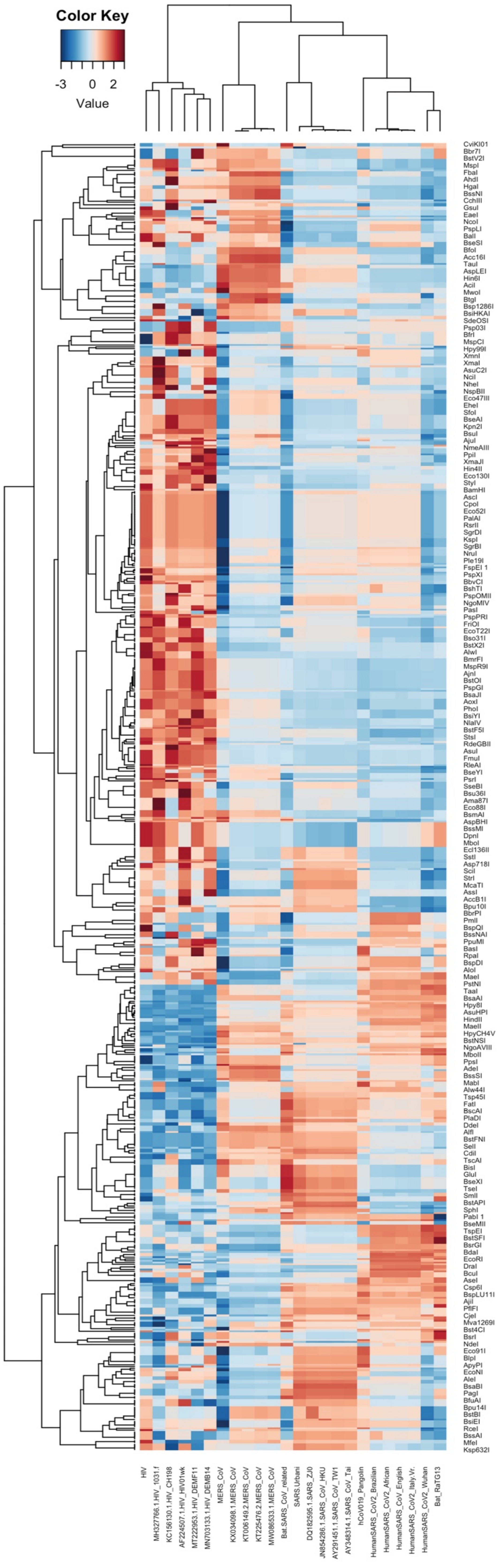

Figure S6. **RS barcodes map in high resolution and full size.** This image is the same image of the Figure 2A and can be usefully to read the labels of the RSs reported on the right of the image.

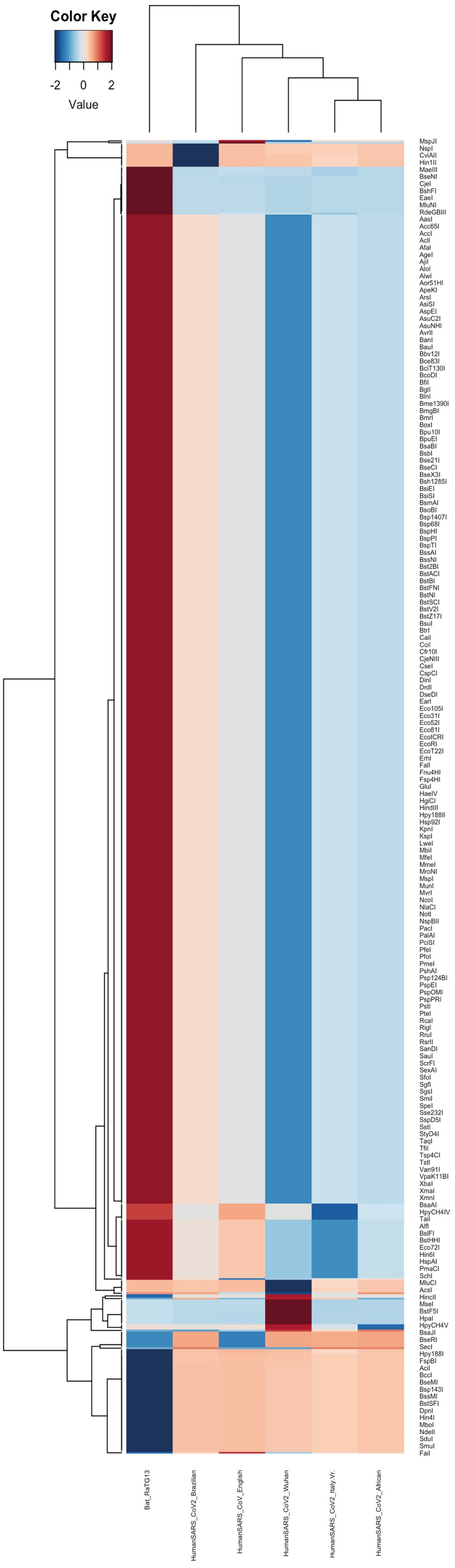

Figure S7. 300 bp RSs barcode map in high resolution and full size. 300 bp. This image is the same image of the Figure 2B and can be usefully to read the labels of the RSs reported on the right of the image.

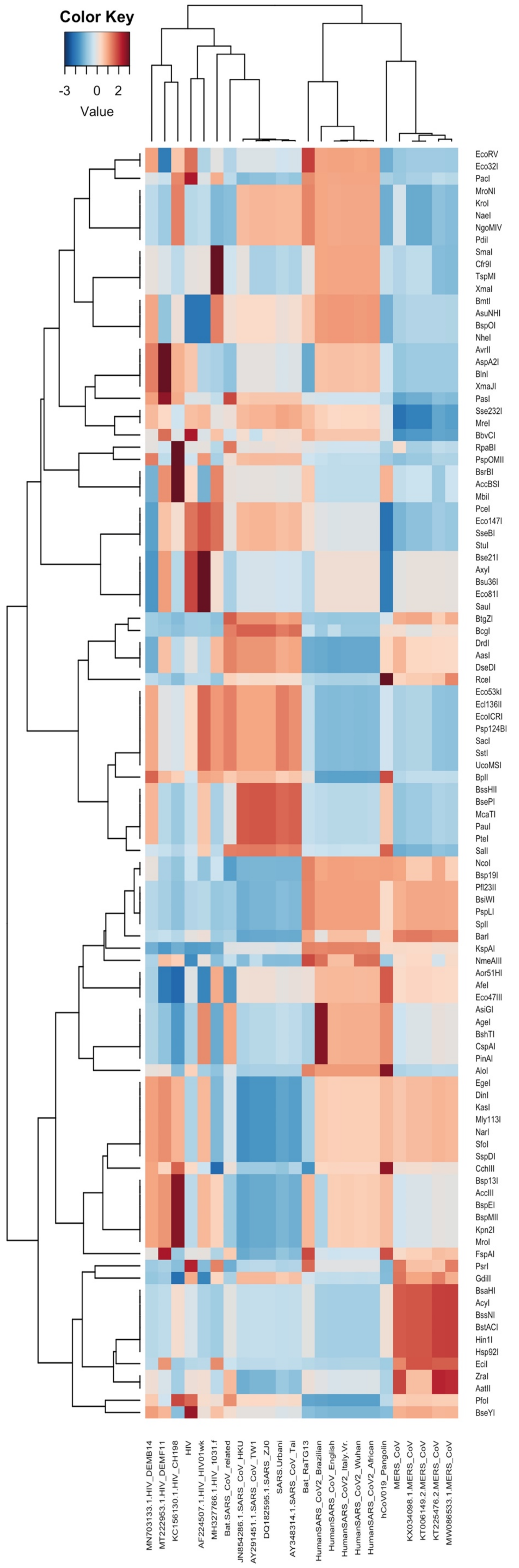

Figure S8. Informative RSs barcode map in high resolution and full size. This image is the same image of the Figure 2D and can be usefully to read the labels of the RSs reported on the right of the image.

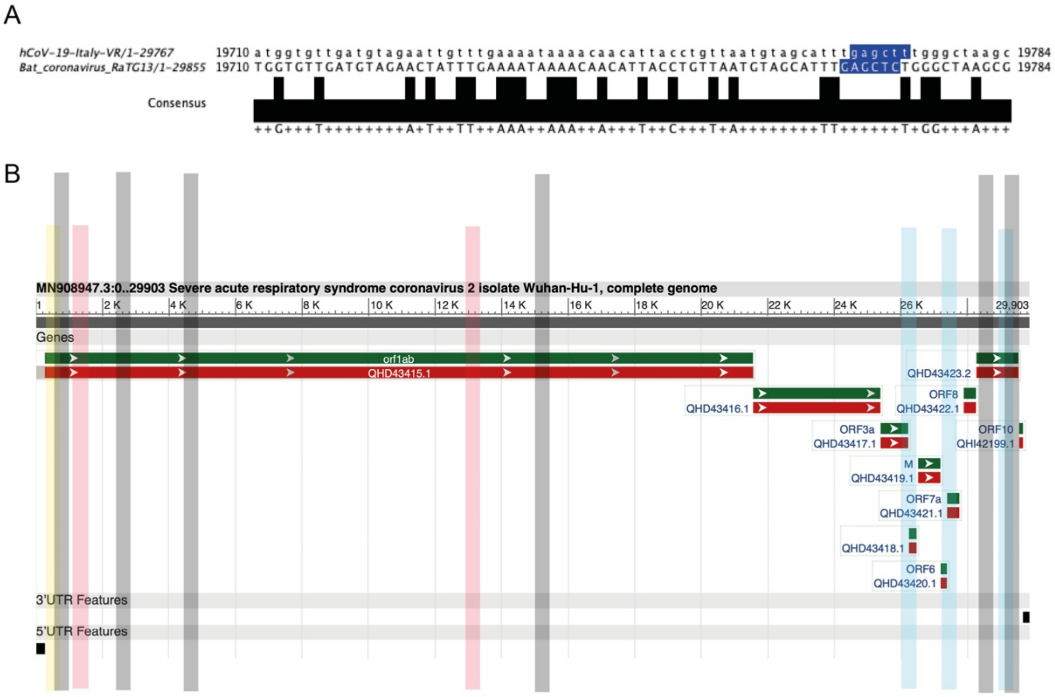

Figure S9. Genomic organization of the SARS-CoV-2 ORFs. The image shows the unique RSs position found in these genomes. Grey bars show position of the unique RSs of the Human SARS-CoV-2, blue bars show the shared RSs found in the last part (20000-30000bp) of the genome, the pink bars show the shared RSs in the middle region (400-20000bp) of the genome and the yellow bar show shared RS in the first part (0-400bp) of the genome.

Supplementary Tables

Tables S1

	icSARS-C7 sequence					SARS-CoV Urbani sequence				
	GCCATAATGGC	GCCAGCGTGGC	TGCCCAAGAGGC	GCCCTCCTGGC	GCCTACACGGC	GCCATAATGGC	GCCAGCGTGGT	TGCCCAGGAGGC	GCATTGCTTGC	GCCTACACTGC
hCoV-19-Italy-Vr	not found	not found	not found	not found	not found	not found	8764	not found	not found	not found
Sars-CoV-19 Wuhan	not found	not found	not found	not found	not found	not found	8781	not found	not found	not found
hCoV-19 Pangolin	not found	not found	not found	not found	not found	not found	not found	not found	not found	not found
Bat CoV RaTG13	not found	not found	not found	not found	not found	not found	not found	not found	not found	not found

Table S1. BglI sites in different virus genomes. The table summarize BglI restriction sites in recombinant icSARS-C7 clone and in wild type SARS-CoV Urbani and compare these sequences in four different SARS-CoV-2 genomes.

Tables S2

BglI restriction sites			
Mutant Sars cov Urbani MA15 SHC014 spike	hCoV 19 Italy VR	Sars CoV 19 Wuhan	Bat-Cov-raTG13
12.366 bp	28.944 bp	29.063 bp	/
6.854 bp	823 bp	840 bp	825 bp
4.330 bp			
3.362 bp			
2.816 bp			

Table S2. Genomic position of the BglI restriction sites. As expected, most of these sites are present in the mutant recombinant viruses. The only two BglI sites present hCoV-19-Italy-VR and in the SARS-CoV-19 Wuhan are located in a different genomic area compared to the recombinant viruses. The BglI site at +/-820 bp was also found in the Bat-CoV-raTG13, suggesting once again the similarity between these two viruses and a possible shared ancestor.
